# Mechanistic divergences of endocytic clathrin-coated vesicle formation in mammals, yeasts and plants

**DOI:** 10.1242/jcs.261847

**Published:** 2024-08-20

**Authors:** Alexander Johnson

**Affiliations:** ^1^ Division of Anatomy, Center for Anatomy & Cell Biology, Medical University of Vienna, Vienna 1090, Austria; ^2^ Medical Imaging Cluster (MIC), Medical University of Vienna, Vienna 1090, Austria; ^3^ Biosciences, University of Exeter, Exeter EX4 4QD, UK

**Keywords:** Clathrin-coated vesicles, Clathrin-mediated endocytosis, Endocytic initiation, Coat formation, Membrane bending

## Abstract

Clathrin-coated vesicles (CCVs), generated by clathrin-mediated endocytosis (CME), are essential eukaryotic trafficking organelles that transport extracellular and plasma membrane-bound materials into the cell. In this Review, we explore mechanisms of CME in mammals, yeasts and plants, and highlight recent advances in the characterization of endocytosis in plants. Plants separated from mammals and yeast over 1.5 billion years ago, and plant cells have distinct biophysical parameters that can influence CME, such as extreme turgor pressure. Plants can therefore provide a wider perspective on fundamental processes in eukaryotic cells. We compare key mechanisms that drive CCV formation and explore what these mechanisms might reveal about the core principles of endocytosis across the tree of life. Fascinatingly, CME in plants appears to more closely resemble that in mammalian cells than that in yeasts, despite plants being evolutionarily further from mammals than yeast. Endocytic initiation appears to be highly conserved across these three systems, requiring similar protein domains and regulatory processes. Clathrin coat proteins and their honeycomb lattice structures are also highly conserved. However, major differences are found in membrane-bending mechanisms. Unlike in mammals or yeast, plant endocytosis occurs independently of actin, highlighting that mechanistic assumptions about CME across different systems should be made with caution.

## Teaser

**Summary:** A Review highlighting some of the mechanistic similarities and differences in CCV formation via CME from mammals, yeast and plants.

## Introduction

Clathrin-coated vesicles (CCVs) are small spheres of membrane ‘coated’ by the protein clathrin. As the end product of the fundamental eukaryotic cellular process of clathrin-mediated endocytosis (CME), they carry extracellular and plasma membrane-bound cargoes from the cell surface into the cell to be processed, and thus are key organelles in many trafficking pathways (McMahon and Boucrot, 2011). Coated vesicles were first observed in 1964 in electron microscopy (EM) images of mosquito oocytes (Roth and Porter, 1964), it was not until 1975 that coated vesicles were isolated from pig brain and the coat protein was identified as clathrin (Pearse, 1975). Since this discovery, the fine details of CCV formation via CME have been well characterized in mammalian systems (Kaksonen and Roux, 2018). However, before the cell biology tools that enabled these advancements in mammalian cells were readily available, yeast (particularly *Saccharomyces cerevisiae*) were used widely to study CME. Established genetics and genome editing tools in yeast allowed for the rapid screening of mutants to identify crucial CME components, and at least 65 endocytosis accessory proteins (EAPs) that are highly conserved between yeast and mammals have been identified (Merrifield and Kaksonen, 2014). This, and extensive subsequent work, has provided a high level of characterization of yeast CME that is comparable to that in mammals (Weinberg and Drubin, 2012).

**Fig. 1.**
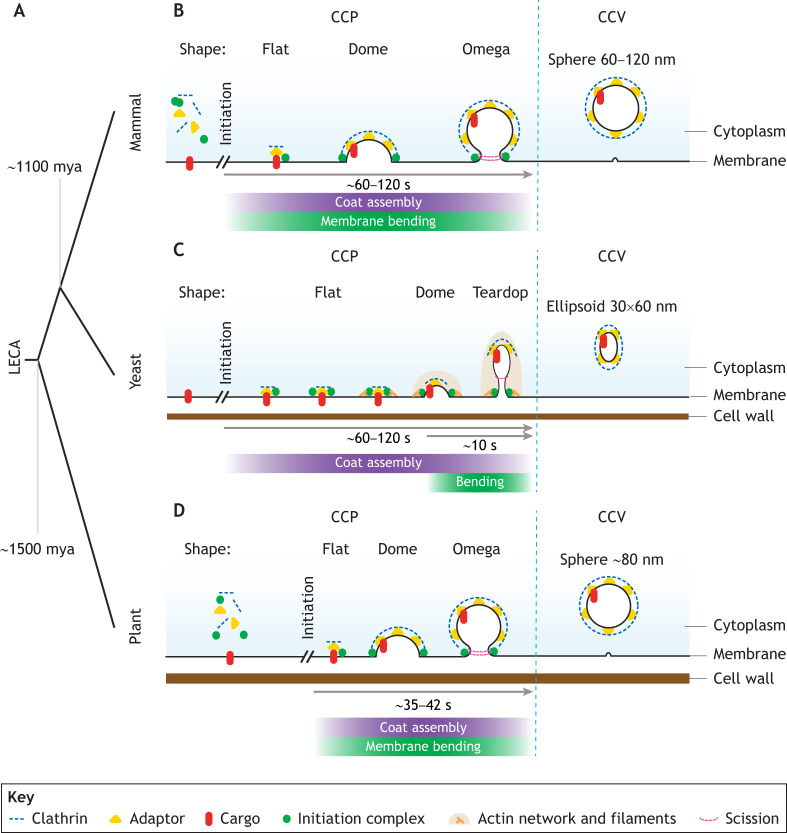
**Overview of mammalian, yeast and plant CME.** Schematics showing (A) the evolutionary time scales of separation of the plants, yeast and mammals from the last common eukaryotic ancestor (LECA). The canonical models for CME reactions for (B) mammals (*Homo sapiens*), (C) yeast (*Saccharomyces cerevisiae*) and (D) plants (*Arabidopsis thaliana*). During CME in each model system, the plasma membrane undergoes remodeling that shapes a flat area of membrane into a CCV, passing through a series of mechanistic steps. These include ‘initiation’, ‘coat assembly’, ‘membrane bending’ and ‘scission’. However, there are differences between the systems, including in temporal ordering of the whole reaction and mechanistic steps, protein machinery and the morphology of the CME structures. For example, coat assembly and membrane bending in the canonical mammalian and plant CME models are coupled, whereas in yeast they are not. Similar morphological shapes of the CME structures (i.e. dome- and omega-shaped CCPs and spherical CCVs) occur in mammals and plants, but differ in yeast, which produce teardrop-shaped CCPs and ellipsoid CCVs.

But how well is CME understood in other eukaryotes? So far, a clathrin gene has been found in every eukaryotic genome sequenced except one (microsporidians) (Robinson, 2015), and CME has been described in a wide range of eukaryotic model organisms, including *Caenorhabditis elegans* (Grant and Hirsh, 1999), *Drosophila melanogaster* (Chen et al., 1991) and *Dictyostelium discoideum* (Vines and King, 2019). However, endocytosis has been less studied in organisms outside of the mammalian and yeast phylogenetic branches. An important example is our understanding of CME in Planta. Despite plant biology being directly relevant to human health, and CME being fundamental to plant physiology (Paez Valencia et al., 2016; Chen et al., 2011; DellaPenna, 1999), only recently has CME in plants undergone detailed examination comparable to that for CME in mammalian and yeast models. These advances have largely been in parallel with the development and optimization of quantitative imaging tools that have enabled us to visualize plant endocytosis at resolutions routinely used in mammals and yeast, including total internal reflection fluorescence microscopy (TIRF-M) to examine live single CME events (Johnson et al., 2020; Taylor et al., 2011; Picco et al., 2015), and EM protocols to directly visualize CCVs and CME structures (Heuser, 1980; Johnson et al., 2022; Morris et al., 2019). Potential EAP orthologs in plants are still being identified (for full lists of potential orthologs, please see Chen et al., 2011; Kraus et al., 2024; Fan et al., 2015). By studying fundamental processes, such as CME in plants (part of the Archaeplastida supergroup), which separated over 1.5 billion years ago from the Amorphea supergroup (containing mammals and yeast) (Fig. 1A), one can gain a wider perspective on diversity of mechanisms involved in eukaryotic cell biological processes. Furthermore, as plant cells possess distinct biophysical parameters compared to those in mammalian and yeast cells, they can provide unique evolutionary and mechanistic insights into endocytosis.

This Review will explore the mechanistic similarities and differences in CCV formation via CME that have been identified in plants, with the aim to shed light on the core principles and proteins involved in CME across the tree of life. Specifically, we will highlight recent advancements using the plant model organism *Arabidopsis thaliana* and how CME in *Arabidopsis* compares to CME in yeast (*S. cerevisiae*) and mammalian models (human and rodent) with a particular focus on the initiation, clathrin coat assembly and membrane-bending mechanisms of CME.

## Mechanistic overview of CCV formation during CME in mammals, yeasts and plants

Although the overall mechanism of CME in the different systems follows a similar stepwise progression (Box 1), there are some subtle differences in mechanisms and the intermediate endocytic structures and morphologies, which are explored below.

**Box 1. Overview of the common steps of CME and the clathrin lattice**

Common features of the CME process can be observed in mammalian, yeast and plant systems. Each system has conserved the same essential steps required to create CCVs: these include initiation, coat assembly, membrane bending and scission. Typically, once CME is initiated, clathrin and the coat EAPs are recruited and assemble the clathrin coat, which can rearrange throughout the duration of CME and cover the growing vesicle. The plasma membrane is also remodeled throughout CME. Membrane-bending EAPs drive an invagination into the cell, acting against opposing intracellular forces, to create CCPs. Once the CCP has reached the size of a mature CCV, EAPs involved in membrane cutting (scission) are recruited to free the CCV from the plasma membrane for subsequent processing within the cell. However, there are significant differences in the temporal coordination, mechanisms and proteins driving these steps between mammal, yeast and plant CME (see Fig. 1B–D).

Perhaps the biggest common feature in CME is the clathrin coat, which appears in the same stereotypical honeycomb pattern and is comprised of the same proteins in each system. Clathrin, which appears to be essential for survival in multicellular organisms (Bazinet et al., 1993), forms the 3D coat structures that surround CCPs and CCVs. These coats are contain clathrin heavy chain (CHCs) and the smaller clathrin light chain (CLCs) proteins. Three CHCs form a triskelion (Greek for ‘three-legged structure’), which act as the building blocks for the coat, and can include but does not require CLCs. Three CHCs form a triskelion with their tripod helix domains, and multiple triskelia join to form a clathrin lattice. The CHC ‘legs’ wrap around the ‘legs’ of other triskelia to form pentagonal, hexagonal, and rarely heptagonal faces (Ungewickell and Branton, 1981) (see figure below). Euler's formula dictates that a closed sphere of polygons must be composed of 12 pentagons (Jin and Nossal, 1993); thus, variations in clathrin coat size and shape are generated by differential arrangement of the clathrin polygons to coat different membrane morphologies during CME. Images below modified from Paraan et al., 2020 (left and right) and Yang et al., 2022 (middle) where they were published under a CC BY 4.0 license.
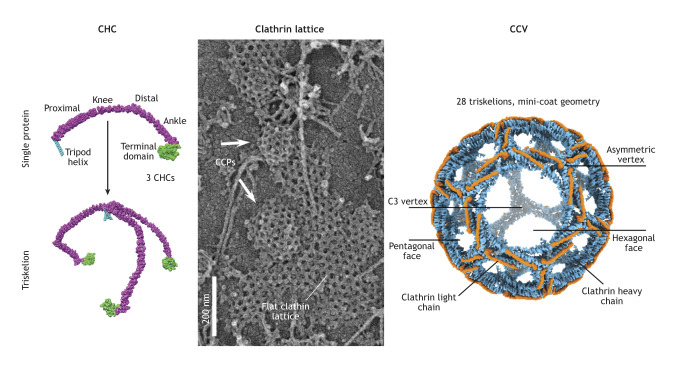

Mammalian CCV formation via CME has been well characterized by a range of experimental approaches (e.g. *in vitro* reconstitution assays, and in cells, tissues and organoids) (Dannhauser and Ungewickell, 2012; Mund et al., 2023; Schoneberg et al., 2018; Aguet et al., 2016). The ‘canonical’ CME process follows the common CME steps (Box 1) and creates stereotypical CME morphologies: the initially flat membrane transitions into a ‘dome’-shaped clathrin-coated pit (CCP), then into an ‘omega’-shaped CCP and finally into a spherical CCV (Fig. 1B). At present, the precise details of how clathrin assembly and membrane bending are temporally coordinated are subject to an ongoing debate, with some reports finding that clathrin assembles prior to membrane bending during CCP growth (see the ‘Making a coat’ section below for further details). The whole CME reaction is reported to take 60–120 s and produces spherical CCVs with a diameter of 60–120 nm, depending on which cell type is examined (Aguet et al., 2013; Taylor et al., 2011; Jaqaman et al., 2008; Kaksonen and Roux, 2018; McMahon and Boucrot, 2011). Not all CME events are productive; for example, in BSC1 cells only ∼39% of CME events successfully produced CCVs and two short-lived, non-productive CME populations were observed (Loerke et al., 2009). In addition to this, a ‘non-canonical’ mode of CME has been observed in several mammalian cell types where large flat plaques of clathrin act as a base for CCV formation from which smaller areas of the clathrin lattice can break off from the plaque edges to form CCVs (Willy et al., 2021; Lampe et al., 2016). Interestingly, non-canonical mammalian CME is dependent on actin and relies on certain clathrin adaptor proteins; specifically, the clathrin adaptor AP2, which links the clathrin plaques to the plasma membrane, and clathrin assembly lymphoid myeloid leukemia protein (CALM), which mediates CCV budding at the plaque edges (Leyton-Puig et al., 2017; Willy et al., 2021; Elkhatib et al., 2017; Saffarian et al., 2009).

In yeast, the CME model is largely based upon imaging endocytosis in the intact single cell organism. The overall mechanism follows the same multi-step progression as the mammalian canonical model (Box 1), but with some fundamental differences (Fig. 1C). Once CME is initiated, the clathrin coat begins to assemble on the flat membrane, which remains flat for 50–110 s (Weinberg and Drubin, 2012). In the last 10 s of yeast CME, membrane bending occurs rapidly in parallel with a burst of actin activity, creating the CCP (Sirotkin et al., 2010). However, these CCPs appear as elongated teardrop-shaped tubules, which are only partially covered with clathrin on their cap (Mund et al., 2018). The scission EAPs then cut the membrane to create ellipsoid CCVs that are ellipsoids of 30 by 60 nm (McMahon and Boucrot, 2011; Kukulski et al., 2012). In contrast to what occurs in mammals, once clathrin is recruited to a CME site in yeast, it is reported that no clathrin-positive CME sites became abortive (Kaksonen et al., 2005). Thus, despite sharing over 65 conserved EAPs and the same stepwise progression of CME (Kaksonen and Roux, 2018; Merrifield and Kaksonen, 2014), clear mechanistic differences between mammals and yeast are present. Interestingly, it has been suggested that this is a consequence of the differing biophysical parameters of the cells, such as turgor pressure and membrane tension, which are higher in yeast than in mammalian cells (Dmitrieff and Nedelec, 2015; Ma and Berro, 2021).

As plant cells are subjected to levels of turgor pressure equal or higher than those in yeast, with reports of ∼0.6 MPa in yeast (Schaber et al., 2010) and up to 2 MPa in plants depending on the cell type (Beauzamy et al., 2014), it was initially anticipated that plant CME would more closely resemble yeast CME. However, based on more recent studies that have imaged intact organs and protoplasts, the overall mechanism of CME in *Arabidopsis* appears to more closely resemble that in mammals, specifically in terms of intermediate morphologies, which could potentially be indicative of similar molecular mechanisms (Fig. 1D). After initiation, coat assembly and membrane bending occur throughout the CME process, producing dome- and omega-shaped CCPs (Narasimhan et al., 2020; Dhonukshe et al., 2007; Li et al., 2012). Scission then occurs, creating spherical CCVs with a diameter of ∼80 nm (Johnson et al., 2022). Population analysis of plasma membrane-localized clathrin in intact roots has revealed that there are three populations of CME events; the productive population, with a lifetime of 42 s, represents 44% of events (Narasimhan et al., 2020). Interestingly, in hypocotyl cells (found in the embryonic stem of a germinating plant), which experience higher levels of turgor pressures than root cells, CME appears to occur faster, at ∼35 s (Narasimhan et al., 2020).

Surprisingly, although plants are evolutionarily further away from mammals than yeast and although plant and yeast cells share some important biophysical properties (e.g. high turgor pressure), the mechanism of CCV formation via CME in plants thus overall appears have more in common with mammalian canonical models of CME. This argument is based upon the order of endocytic steps, the morphologies of CME events and CCVs (i.e. existence of omega-shaped CCPs and spherical CCVs rather than the teardrop-shaped CCPs and ellipsoid CCVs found in yeast) and relative populations of productive versus abortive events. However, evidence for a non-canonical CME is thus far absent in yeast and plants, as large clathrin plaques have not been observed in either system. Next, we will compare what is known regarding the specific proteins involved in the progressive CME steps in mammals, yeast and plants.

## Initiation of endocytic CCV formation

**Fig. 2.**
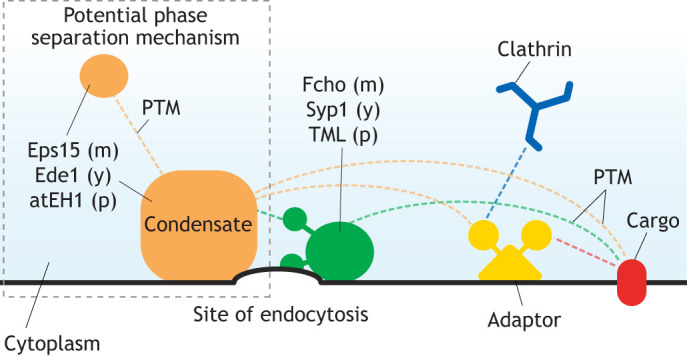
**A potential common mechanism of endocytic initiation.** A schematic of a potential common initiation mechanism for CME in mammals (m), yeast (y) and plants (p). Dotted lines represent interactions between components of the initiation machinery. EH domain-containing proteins shown in orange [Eps15 (m), Ede1 (y) and atEH1 (p)], are reported to be key initiators of endocytosis and can interact with the membrane and other important initiation factors, notably µ homology-containing domain proteins, shown in green [Fcho (m), Syp1 (y) and TML (p)]. Together, these proteins then interact with clathrin adaptor proteins to drive clathrin assembly. Furthermore, the EH domain-containing proteins can also drive cargo selection directly, as they can bind cargoes marked for internalization. This activity is mediated by PTM processes like ubiquitylation. Shown in the gray dotted box is phase separation, regulated by PTMs like phosphorylation, which might also modulate the role of the EH domain-containing proteins at endocytic sites. However, the involvement of phase separation in initiation of CME is subject to on-going debate.

To date, a small number of EAPs have been identified as mediators of endocytic initiation. These are recruited at the beginning of CME and generate a platform for the CME reaction by beginning to deform the plasma membrane and can also play roles in cargo selection (Fig. 2).

In mammals, these EAPs include FCH domain only proteins (Fcho1 and Fcho2) and epidermal growth factor receptor substrate 15 (Eps15) (Henne et al., 2010; Taylor et al., 2011; Wang et al., 2016). Deletion and disruption of these factors significantly increases the number of abortive CME events (Ma et al., 2016; Wang et al., 2016; Carbone et al., 1997; Benmerah et al., 1999). Fcho1 and Fcho2 contain an F-BAR domain, which can both sense and generate membrane curvature as a result of its curved conformation and through insertion of its N-terminal amphipathic helix into the membrane, facilitating accumulation of these proteins at CME sites (Shimada et al., 2007; Qualmann et al., 2011; Henne et al., 2007). Additionally, they contain a µ homology domain, which can bind Eps15 (Ma et al., 2016; Cupers et al., 1997). Eps15 can also insert into the membrane to generate membrane curvature (Wang et al., 2016; Daumke et al., 2007). It is thought that the molecular crowding effect caused by accumulation of Fcho and Eps15 molecules at CME events can also contribute to membrane deformation (Stachowiak et al., 2012). In addition, Eps15 has been reported to bind ubiquitylated cargoes (Polo et al., 2002), and can thus mediate cargo selection. However, as the endocytosis reaction progresses, Fcho and Eps15 are restricted to the rims of the clathrin assembly area and are left behind once the CCV departs the plasma membrane (Sochacki et al., 2017).

The yeast homologs to Eps15 and Fcho1 and 2 are EH domains and endocytosis 1 (Ede1), and suppressor of yeast profilin 1 (Syp1), respectively. Ede1 contains Eps15 homology (EH) domains and Syp1 contains both an F-BAR and µ homology domain (Kaksonen and Roux, 2018). They too are recruited early at sites of CME (Picco et al., 2015) and surround the clathrin assembly (Mund et al., 2018). As in mammals, their deletion results in a significant decrease in productive endocytosis events (Kaksonen et al., 2005; Carroll et al., 2012). These proteins also appear to have a role in cargo selection, as their deletion results in defective cargo recruitment (Brach et al., 2014). Together, these findings suggest that these proteins perform similar functions to their mammalian homologs.

Plants also possess proteins in which some of these key ‘initiation’ domains are conserved: *Arabidopsis thaliana* (at) protein, atEH1/pan1 and atEH2/pan1 (hereafter referred to as atEH for simplicity), containing EH domains, and TPLATE complex muniscin-like (TML), containing a µ homology domain (Gadeyne et al., 2014; Zhang et al., 2015). These proteins are part of the octameric TPLATE complex (TPC), an ancient complex that is conserved in many eukaryotes within the Amorphea supergroup (such as Amoebozoa and Thecamonas, where it is known as the TSET complex), but is absent in mammals and yeasts (Hirst et al., 2014). For a detailed examination of the distribution of the TPC across eukaryotes and its predicted structural homologies, please refer to Hirst et al., (2014), Kraus et al. (2024) and Yperman et al. (2021). In plants, TPC subunits are recruited together early at CME sites (Wang et al., 2020) and are localized to the rim of CME events (outside of the eventual CCV) (Dahhan et al., 2022; Johnson et al., 2021). Deletion of the TPC subunit TPLATE is lethal in *Arabidopsis* (Gadeyne et al., 2014) and its disruption blocks CME by preventing endocytic membrane bending (Johnson et al., 2021). Similar to the interactions between EH domain-containing (Fcho or Syp1) and µ homology domain-containing (Eps15 or Ede1) proteins in mammals and yeast, the µ homology domain of TML links the atEH proteins to the TPC (Yperman et al., 2021). Although the TPC lacks any proteins with an F-BAR domain, the µ homology domain of TML has been reported to bind directly to the membrane, and the EH domains of the atEH proteins have intrinsic membrane bending activity, similar to Eps15 (Yperman et al., 2021; Johnson et al., 2021), which might substitute for the function of the F-BAR domain in the other systems. The TPC has also been reported to drive the recruitment of cargo during endocytosis (Grones et al., 2022; Sanchez-Rodriguez et al., 2018; Bashline et al., 2015; Wang et al., 2023). Furthermore, overexpression of atEH1 and artificial relocalization of TPLATE to mitochondrial membranes has been reported to nucleate clathrin assembly (Dragwidge et al., 2024). However, it is interesting to note that clathrin assembly on the plasma membrane can occur without TPLATE or an intact TPC (Johnson et al., 2021). Together, these data suggest that plants might rely upon this ancient complex to initiate CME while also making use of EAPs containing EH and µ homology domains in a similar fashion to both mammals and yeast.

In further support of the hypothesis that these conserved domains provide a common initiation mechanism for CME across the tree of life is that, in each system, their function and regulation has been reported to depend on a common process: phase separation of the EH domain-containing EAP (Eps15, Ede1 or atEH1) (Dragwidge et al., 2024; Day et al., 2021; Kozak and Kaksonen, 2022). Furthermore, as post-translational modifications (PTMs), such as phosphorylation, are thought to regulate the formation of condensates in phase separation (Li et al., 2022), it is interesting to note that Eps15, Ede1 and atEH1 are all phosphorylation substrates. Eps15 phosphorylation mutants produce disruptions in CME (Confalonieri et al., 2000), Ede1 is one of the most phosphorylated proteins in yeast (Lu et al., 2016) and phosphorylation of specific sites on the atEH proteins in response to stress might alter endocytic processes (Jain and Schmidt, 2024). Therefore, this key step in CME appears to be well conserved, as it relies on conserved protein domains and potentially utilizes a similar phase separation mechanism regulated by PTMs to control EH domain-containing protein function (Fig. 2). However, as Opisthokonts (including mammals and yeast) lack most of the genes encoding the ancient TPC, they appear to have evolved to use F-BAR domain-containing proteins to replace the TPC function (Hirst et al., 2014). This key evolutionary difference highlights the utility of comparing a range of model systems in improving our understanding of the evolution and mechanisms of CME.

## Clathrin coat assembly

### Meet the clathrins

**Fig. 3.**
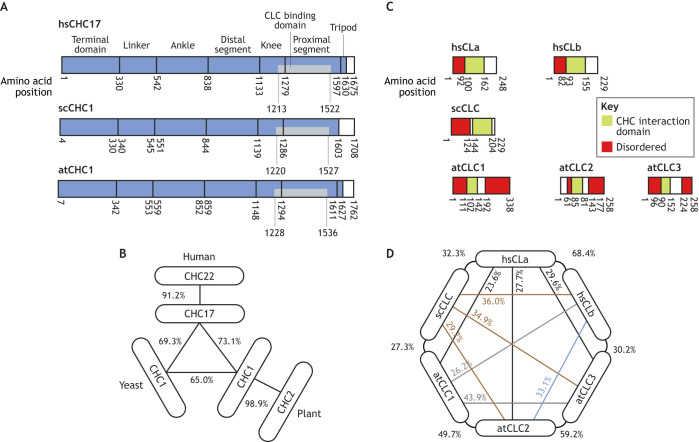
**Sequence similarities of the clathrin proteins and the process of lattice formation.** (A) Schematic representations of the clathrin heavy chain proteins from *Homo sapiens*, *Saccharomyces cerevisiae* and *Arabidopsis thaliana* and (B) their computed sequence similarities. CHCs in each species contain key conserved domains and generally have high sequence similarity. (C) Schematic representations of the clathrin light chain proteins from *H. sapiens*, *S. cerevisiae* and *A. thaliana* and (D) their sequence similarities. CLCs in each species possess two conserved domains; however, plant CLCs contain an additional C-terminal disordered domain not found in human or yeast CLCs. CLC sequence similarity between species is less conserved than in CHCs. scCHC and atCHC domains were defined using NIH BLAST (https://blast.ncbi.nlm.nih.gov/blast/Blast.cgi), with the *Homo sapiens* domains as a reference. Sequence similarities were determined using EMBOSS Needle (Madeira et al., 2022).

Here, we will compare clathrin proteins found in plants, yeast and mammals, using human clathrins as a representative example. See Box 1 for a general overview of clathrin coat assembly and structure. In *Homo sapiens* (hs), there are two CHCs (hsCHC17 and hsCHC22; also known as CLTC and CLTCL1, respectively) and two CLCs (hsLCa and hsLCb; also known as CLTA and CLTB, respectively). hsCHC17 is expressed in all cell types and has an essential role in forming CCVs at the plasma membrane (Dannhauser et al., 2017). Its domains and structure are well defined. Key regions are the terminal domain (a globular region at the N-terminus), the distal segment, the proximal segment (where CLCs bind) and a C-terminal tripod helix, which mediates CHC trimerization to form the triskelion (Fotin et al., 2004) (Fig. 3A). Although hsCHC22 shares 91% sequence similarity with hsCHC17 (Fig. 3B), they play different roles in mammalian physiology. It is interesting to note that CHC22 is found in most tetrapods, but has become a pseudogene in mice and other rodents (Fumagalli et al., 2019; Wakeham et al., 2005). In contrast to the ubiquitous expression and essential role of hsCHC17, hsCHC22 is limited to muscle cells, adipocytes and transient expression during neuronal development (Nahorski et al., 2015; Vassilopoulos et al., 2009). Furthermore, it has been reported that hsCHC22 does not form CCVs via CME at the plasma membrane (Dannhauser et al., 2017), and that it does not associate with CLCs (Liu et al., 2001).

The hsCLCs contain two major domains: a disordered N-terminal domain and an α-helical CHC interaction domain (Fig. 3C). hsLCa and hsLCb share 60% sequence similarity and although the hsCLCs have been implicated in a range of cellular and developmental processes (Das et al., 2021), much less is known about their precise physiological roles. However, knockout and knockdown of hsCLCs has been found to cause receptor trafficking defects (Majeed et al., 2014; Poupon et al., 2008; Wu et al., 2016), and it is thought that specific interactions of hsCLCs with hsCHC17 can drive different clathrin-mediated processes (Redlingshofer et al., 2020). Interestingly, it has been reported that CLC stabilizes hsCHC17 triskelia (Fotin et al., 2004), which alters the biophysical properties of clathrin assemblies (Dannhauser et al., 2015), suggesting that specific combinations of CHCs and CLCs could be required to produce CCVs under differing biophysical constraints in different mammalian cell types.

*S. cerevisiae* (sc) express a single CHC (scCHC1) and CLC (scCLC1). Although scCHC1 is highly conserved with hsCHC17, possessing 69% sequence similarity and the same organization of domains (Fig. 3A,B), it was originally reported to not be essential in yeast, as its deletion produced viable cells albeit with slowed growth (Payne et al., 1988). However, later work suggested a key role for clathrin in yeast and implicated it in the early organization of endocytic events: expression of temperature-sensitive scCHC1 mutants resulted in a 30–50% reduction in endocytosis compared to that in wild-type cells (Tan et al., 1993), and deletion of scCHC1 resulted in severe delays in early endocytic patch formation (Newpher and Lemmon, 2006). It has more recently been suggested that scCHC1 regulates CCV size rather than controlling the overall endocytic reaction (Kukulski et al., 2016). scCLC1 also retains the same domains and organization as the hsCLCs, but it only shares 32.2% sequence similarity with them (Fig. 3C,D). Deletion of scCLC1 produces similar phenotypes to those found in sc*chc1* deletion mutants and results in a reduction of CHC on the cell surface (Chu et al., 1996; Silveira et al., 1990). It is thought that scCLC1 provides a crucial link between the clathrin coat and actin, which can in turn mediate yeast endocytic progression (Boettner et al., 2011).

*Arabidopsis thaliana* has two CHCs (atCHC1 and atCHC2) and three CLCs (atCLC1, atCLC2 and atCLC3). atCHC1 and atCHC2 are almost identical, with a sequence similarity of 98.9%, and are ∼70% similar to hsCHC17, and ∼65% similar to scCHC, and contain the same domains and organization as human and yeast CHCs (Fig. 3A,B). Plants with deletion of both atCHCs are not viable, and inducible overexpression of a dominant-negative CHC in seedlings has been found to inhibit CME, resulting in severely disrupted growth (Kitakura et al., 2011). Although it has been reported that the atCHCs are functionally redundant and share the same subcellular localization patterns, subtle developmental defects in embryos and leaves have only been reported in the at*chc2* mutants (Kitakura et al., 2011).

In contrast to atCHCs, the atCLCs display some significant divergences from the human and yeast CLCs. The disordered N-terminal and the CHC interaction domains are conserved; however, an additional disordered domain is present at the C-terminal (Fig. 3C). atCLC2 and atCLC3 are 60% similar to each other and show ∼30% sequence similarity to the human and yeast CLCs, but atCLC1 shows only 44% and 50% similarity to atCHC2 and atCHC3, respectively, and just ∼25% sequence similarity to the yeast and human CLCs (Fig. 3D). Deletion of the atCLCs produces developmental phenotypes: for example, at*clc2* and at*clc3* single mutants, as well as at*clc2* at*clc3* double mutants all have shorter roots and hypocotyls than are seen in wild-type plants (Wang et al., 2013). Most notably, at*clc1* mutants are not viable (Wang et al., 2013; Konopka et al., 2008). Interestingly, when colocalization of atCHC1 and atCLC2 was examined on the plasma membranes of root cells, the co-incidence rate was 62% (Narasimhan et al., 2020). Together, these findings suggest that, as in mammalian cells, different clathrin combinations might regulate specific clathrin-mediated physiological processes in plant cells.

In summary, given the essential role of clathrin in both cell and organismal physiology, it is perhaps no surprise that the CHCs are highly conserved across mammals, yeast and plants, displaying almost identical domain organization in each system. However, more variation is found in the CLCs. In particular, atCLC1 significantly diverges from both its other atCLC counterparts and yeast and human CLCs, and its function cannot be compensated for by atCLC2 or atCLC3 (Wang et al., 2013).

### Making a coat

The details of how clathrin polygon arrangements can form a coat were described in mammalian cells in the 1980s (Heuser, 1980), but researchers have now described the clathrin lattice at atomic resolutions (Box 1). hsCHC can assemble clathrin cages *in vitro*, and cryo-electron microscopy (cryo-EM) has been used to resolve the molecular details of reconstituted CCVs. Using this method, several stereotypical clathrin coats made of specific combinations of polygonal faces have been described (Fotin et al., 2004; Morris et al., 2019; Kirchhausen and Harrison, 1981). Similar approaches have also been used to examine natively assembled CCVs, thus enabling examination of clathrin coats of fully loaded vesicles and their CCV-associated proteins. Native CCVs were found to be more heterogenous in their polygon arrangements (Paraan et al., 2020). Together, these approaches have also uncovered that adaptor proteins, which provide the physical link between clathrin and the plasma membrane, can influence the formation of different clathrin geometries (Fotin et al., 2004; Paraan et al., 2020; Kovtun et al., 2020).

How the coat dynamically forms around the growing CCP in CME to create a CCV is an area of much debate. There are two main theoretical models, both of which focus on a link between coat formation and membrane bending (see the ‘Endocytic membrane bending in CCV formation’ section for more information on bending mechanisms) (Kaksonen and Roux, 2018). The first model, called the ‘constant area’ model, hypothesizes that clathrin initially assembles as a flat patch on an area of the membrane and remodels into a spherical cage through a continuous exchange of clathrin molecules. The second model is known as the ‘constant curvature’ model and hypothesizes that the clathrin lattice polymerizes in synchronization with the growth of the membrane invagination at the forming CCP. There is experimental evidence for both models in mammalian cells. Where results from *in vitro* and reconstitution assay approaches favor the ‘constant curvature’ model (Saleem et al., 2015; Dannhauser and Ungewickell, 2012; Dannhauser et al., 2015), *in vivo* data lends more support to the ‘constant area’ model (Mettlen et al., 2010; Maupin and Pollard, 1983; Avinoam et al., 2015; Sochacki et al., 2021; Bucher et al., 2018). However, *in vivo* evidence for the ‘constant curvature’ model also exists (Willy et al., 2021). Interestingly, the morphological transitions of the membrane (e.g. flat, dome and pit) during endocytosis events from these different models appear to be similar (Sochacki et al., 2021; Bucher et al., 2018; Willy et al., 2021), and it is of course possible that both models of coat assembly are utilized in different cell types and within individual cells (Scott et al., 2018).

At present, the details of how clathrin assembles and aggregates in yeast have not been clarified. While time-resolved electron tomography and super-resolution imaging have captured the dynamic stages of yeast CME (Kukulski et al., 2012; Mund et al., 2018), very few studies have examined the coat itself. Characterization of mammalian clathrin coats has relied upon metal replicas of ‘unroofed’ cells (which exposes the intracellular plasma membrane, allowing the visualization of the intracellular plasma membrane and its associated structures) to enable direct examination, but in yeast, these unroofing techniques have failed to allow visualization of clathrin lattices on the plasma membrane (Rodal et al., 2005). However, the yeast clathrin coat has been found to be first recruited to sites of CME as a flat patch, prior to the late membrane-bending stages of CME (Kukulski et al., 2012; Mund et al., 2018; Kaksonen et al., 2005; Picco et al., 2015), thus suggesting that coat assembly in yeast favors the ‘constant area’ model.

Similarly, the fine structural details of plant clathrin and CCVs have not been resolved to date. Clathrin assemblies have been examined in metal replicas of unroofed plant cells (Traas, 1984; Fowke et al., 1983), but recently a 3D quantitative analysis of these natively assembled structures was conducted using scanning transmission electron microscope (STEM) tomography in both unroofed cells and in isolated CCV preparations from *Arabidopsis* (Johnson et al., 2022). As in mammalian cells, the clathrin coat was made of differing combinations of pentagons and hexagons and produced spherical and regularly sized CCVs. Furthermore, proteomic analysis of plant CCVs has revealed a 1:1 ratio of CHCs to CLCs, indicating that the plant triskelion is also composed of individual CHCs interacting with single CLCs (Dahhan et al., 2022). Based upon studies that observed the dynamic recruitment of fluorescently labeled atCLC2 or examined CCPs in metal replicas of unroofed *Arabidopsis* cells, it has been reported that clathrin assembles as the CCP invaginates, in accordance with the ‘constant curvature’ model (Narasimhan et al., 2020). In support of this, imaging of plant cells in several studies has shown that clathrin accumulates in individual foci on the plasma membrane and that large, flat clathrin plaques are not observed (Johnson and Vert, 2017; Konopka et al., 2008; Johnson et al., 2020; Gadeyne et al., 2014; Fujimoto et al., 2010; Traas, 1984)). It is important to note that in many fluorescence imaging studies of plant clathrin, large ‘blobs’, which might be mistaken for plaques, are visible, but these ‘blobs’ are highly dynamic structures that are thought to represent collections of CCVs at early endosomes, and that CCVs in *Arabidopsis* appear to be uncoated more slowly than in mammals or yeast (Narasimhan et al., 2020).

In summary, although the structure and arrangement of the clathrin lattice is conserved in each system, how the coat assembles is less clear. Mammalian studies report that the coat can form either independently of membrane bending, resembling coat assembly in yeast in which a flat patch is formed before bending occurs, or in parallel with bending, which appears to be how plants form clathrin coats.

## Endocytic membrane bending in CCV formation

Although a variety of mechanisms have been reported to drive endocytic membrane bending, this Review will focus on the potential roles of the coat, as it is present in all systems, and actin, as there is experimental evidence for their involvement in each system.

### Bending driven by the coat

Given that clathrin was clearly and strongly associated with curved membranes in early EM images, it was first proposed that it could function to bend the membrane, giving rise to CCPs and CCVs (Kirchhausen and Harrison, 1981; Pearse, 1975). However, the idea that the coat itself can drive membrane bending has been disputed and debated. One issue with this model is the fact that clathrin cannot directly bind to the membrane, thus suggesting that clathrin might instead act as a scaffold and organizer for the membrane-bending EAPs.

Through *in vitro* reconstitution assays using a minimal set of mammalian components involved in CME, it has been shown that clathrin itself can provide sufficient force to bend membranes (Dannhauser and Ungewickell, 2012). However, this only occurred at low membrane tensions; when tension was increased, additional EAPs – particularly adaptor proteins – were required to induce bending (Saleem et al., 2015; Boulant et al., 2011; Scott et al., 2018). Recently, it has also been shown that clathrin can induce curvature *in vivo* – when clathrin was inducibly re-localized to mitochondrial membranes, which lack many EAPs, CCV formation occurred at the mitochondria (Kuey et al., 2022). Similarly, clathrin has been shown to be recruited to artificially induced membrane invaginations in cells plated on ‘bumpy’ coverslips, where it stabilized and facilitated further membrane curvature (Cail et al., 2022; Kuey et al., 2022). In yeast, however, the coat assembly and membrane bending stages are clearly temporally uncoupled, suggesting that coat assembly does not drive membrane bending in this system (Mund et al., 2023; Kukulski et al., 2012). Further evidence that coat formation and membrane bending are distinct, independent processes is found in mammalian and plant cells in which essential EAPs have been disrupted. In human retinal pigment epithelial cells in which the µ homology domain of AP2 was deleted, flat clathrin assemblies with about the same diameter as CCVs were detected on the plasma membrane (Aguet et al., 2013). Similarly, when the TPC (containing TML, which has a domain that is homologous to the mammalian AP2 µ domain) was disrupted in *Arabidopsis* by destabilizing the complex with expression of an inducible loss-of-function TPLATE protein, flat clathrin patches around the same size as plant CCVs were regularly observed (Johnson et al., 2021).

While the debate continues about the precise role of the clathrin coat in membrane bending, it cannot be excluded that it can both contribute to membrane bending and provide scaffolding and/or organization of EAPs. However, its bending effects have thus far only be seen under specific experimental conditions.

### Bending driven by actin

**Fig. 4.**
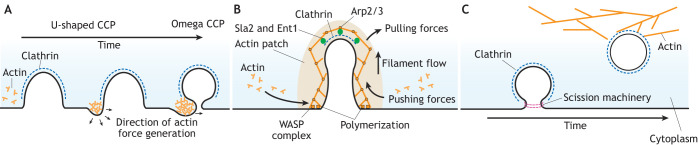
**The role of actin in CME.** Schematics demonstrating proposed roles of actin during CME in mammalian, yeast and plant cells. (A) Although the requirement for actin in CME in mammalian cells is variable, in some cell types, actin localizes asymmetrically at sites of CME, becoming concentrated at one side of the rim of ‘U’-shaped CCPs. Over time, accumulation of actin presses the membrane sideways, closing the neck of the pit and producing the ‘omega’ shape. (B) In yeast, actin is an absolute requirement for driving the membrane invagination inwards against the high turgor pressure. Actin monomers are recruited to cover the clathrin assembly area of an endocytic event via interactions with Sla2 and Ent1, forming an actin patch. Actin then undergoes polymerization (mediated by the WASP and Arp2/3 complexes) at the rim of the endocytic event, producing growing filaments that provide pushing and pulling forces to drive membrane bending and shape the CCP. (C) Actin is not reported to be involved in the plant CME reaction but is involved in mediating post-endocytic trafficking of vesicles once they are freed from the plasma membrane.

Actin filaments are known to produce forces within cells and localize to CME events in mammals and yeasts (Merrifield et al., 2002; Kaksonen et al., 2003; Sirotkin et al., 2005; Taylor et al., 2011); thus, actin is a strong candidate for being the factor that drives invagination of the membrane inwards against opposing intracellular forces during CME. However, the requirement for actin in mammalian endocytic CCV formation is hotly debated, and also appears to vary depending on which cell type is examined (Fujimoto et al., 2000). For example, actin is not required for uptake of transferrin (a canonical CME cargo) in cultures of BSC1 and HeLa cells (Boucrot et al., 2006; Saffarian et al., 2009), but has been reported to be required for its uptake in A431 cells, as treatment with the actin polymerization inhibitor latrunculin A reduced transferrin uptake by 50% (Lamaze et al., 1997). Interestingly, altering certain biophysical properties of cells (i.e. by disrupting the cytoskeleton or increasing membrane tension with hypo-osmotic media) creates a requirement for actin in CME (Boulant et al., 2011). Actin has also been found to be preferentially recruited to stalled CCPs (Jin et al., 2021 preprint), and together these results suggest that actin can indeed provide membrane-bending forces during CME to overcome the effects of elevated membrane tension or turgor pressure. However, in cells where actin is reported to be required for CME, actin and cellular biophysical alterations appear to affect the later stages of endocytosis. Specifically, the abundance and lifetimes of CCPs on the cell surface is notably increased, and these CCPs have a ‘U’-shaped morphology rather than the typical ‘omega’ shape (Boulant et al., 2011; Ferguson et al., 2017). This highlights that although sufficient membrane bending can occur to create a CCP without the participation of actin, actin could be involved in budding the CCV from the plasma membrane. Indeed, live imaging combining atomic force microscopy and confocal microscopy has found that actin asymmetrically accumulates at the rim of CCPs and is responsible for closing the CCP by driving the formation of the ‘omega’ stage (Yoshida et al., 2018) (Fig. 4A). Furthermore, actin dynamics at CME events have been linked to recruitment of dynamin, the canonical scission protein involved in CME (Taylor et al., 2012), further hinting at possible regulatory roles of actin in CCV budding and scission.

In contrast to what is seen in mammals, the requirements for actin in yeast CME is much clearer. Disruption of the actin network blocks CME in yeast (Idrissi et al., 2012; Kukulski et al., 2012; Ayscough et al., 1997; Kaksonen et al., 2003; Moreau et al., 1997). During CME, actin is recruited prior to and at the initiation of membrane bending (Kaksonen et al., 2005), where it first creates a 200 nm cover under the CME event and then polymerizes to cover the invaginated CCP (Idrissi et al., 2012; Kukulski et al., 2012). Importantly, it has been reported that the polymerization of actin is focused largely at the rim of CME events (Mund et al., 2018), and working models suggest that this activity provides both pushing and pulling forces to drive the membrane inwards (Pollard and Borisy, 2003; Carlsson and Bayly, 2014; Dmitrieff and Nedelec, 2015; Serwas et al., 2022) (Fig. 4B). To provide these forces, actin must be linked to both the coat and the membrane. This link is provided by Pan1 and End3, which recruit actin polymerization machinery, such as the Wiskott–Aldrich syndrome protein (WASP) and Arp2/3 complexes, to CME sites (Sirotkin et al., 2005; Sun et al., 2006). Furthermore, epsin proteins and Sla2 (HIP1R in mammals) interact with actin filaments, binding them to the clathrin coat and the membrane (Tonikian et al., 2009; Boulant et al., 2011; Kaksonen et al., 2003; Skruzny et al., 2012, 2015).

As mentioned above, given that plants cells have equal or even higher levels of turgor pressure that must be overcome during CME compared to those in yeast (Beauzamy et al., 2014), it was initially thought that actin would be essential for plant CME. However, efforts to colocalize a range of actin markers with endocytic events at the cell surface found very low rates of co-incidence in *Arabidopsis* root cells (Narasimhan et al., 2020). Assaying the overall efficiency of endocytosis using the membrane label FM4-64 revealed no significant defects of label uptake during chemical disruption of actin activity (Narasimhan et al., 2020). Furthermore, no significant changes in the dynamics or density of clathrin at the cell surface associated with actin inhibition were found (Konopka et al., 2008; Narasimhan et al., 2020). However, actin inhibition caused mislocalization of cargo proteins and halted early endosome dynamics, thus demonstrating that actin has a role in CCV trafficking after endocytosis (Narasimhan et al., 2020) (Fig. 4C). Although these studies focused on *Arabidopsis*, similar FM4-64 uptake experiments in *Chara corallina* (a freshwater plant that diverged from *Arabidopsis* 500 million years ago) also reported no differences in endocytic efficiency associated with actin inhibition (Klima and Foissner, 2008). It is interesting to note that plants do not appear to have a homolog of WASP, an essential component of the endocytic actin nucleation process in yeast (Kurisu and Takenawa, 2009), highlighting a further divergence of the endocytic machinery in plants.

In summary, data from mammalian studies suggests that actin is required to drive membrane bending when higher levels of opposing intracellular forces are present. This idea is strongly supported by the fact that actin is essential for CME in yeast cells, which experience high turgor pressure. However, data from studies in plants, where endocytic membrane bending occurs independently of actin, suggests that plants might have evolved an alternative mechanism to overcome extreme turgor pressure.

## Conclusions and perspectives

Recent advancements in our understanding of the details of CCV formation during endocytosis in plant model organisms have not only provided many key insights into the cellular processes that underlie plant development and physiology but have also given us a new perspective on the core principles of the fundamental eukaryotic process of CME. Although CME shares similar features across mammalian, yeast and plant model systems, there are also some surprising differences (Table 1). For example, even though plant and yeast cells share similar biophysical parameters (such as high turgor pressure), the morphological stages of plant CME appear to resemble those in mammals, despite the greater evolutionary divergence between plants and mammals. Surprisingly, plant CME overcomes extreme turgor pressures independently of actin. In contrast, the early stages of CME and the clathrin coat appear to be highly conserved in all three systems, and each system potentially relies upon the same key domains to initiate CME and produces similar honeycombed CCV structures.

**Table 1.**
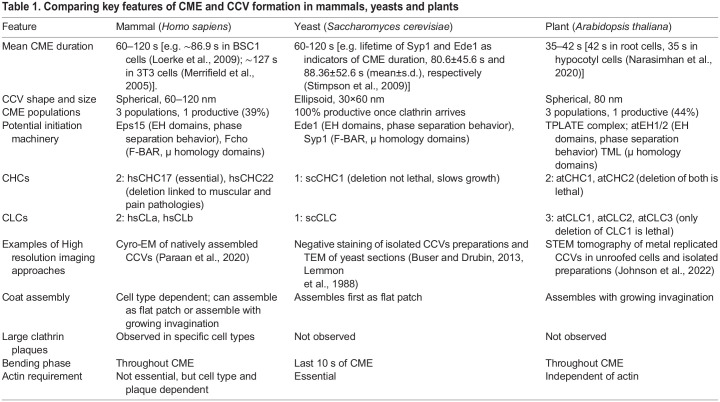
Comparing key features of CME and CCV formation in mammals, yeasts and plants

Going forward, important lessons can be drawn by comparing results from the different approaches used in various experimental model systems. For example, it is clear that the biophysical environment can affect endocytosis mechanisms, so researchers should take care to consider this when interpreting results from specific experimental setups and sample types. For example, although CME in mammals has been extensively examined using a range of experimental approaches including in cultured cells, tissues and organoids, intravital microscopy (IVM) studies have reported different rates of cargo uptake in intact animals compared to cell culture systems (Masedunskas et al., 2012). Furthermore, caution should be taken when making broad mechanistic assumptions about CME based on data from a differing model system. The actin-independent nature of plant CME especially highlights this.

Many exciting open questions about CME across the tree of life remain. As actin is not required for membrane bending in plants, what factor is driving it? TPLATE can mediate membrane bending (Johnson et al., 2021), but whether further unidentified proteins and mechanisms might be involved in generating sufficient force to overcome the extreme turgor pressure are significant open questions. Additionally, is phase separation a master orchestrator of endocytosis? Phase separation is increasingly implicated in cell biological processes; however, it is extremely technically challenging to demonstrate phase separation *in vivo* with native levels of proteins at endocytic scales (<200 nm). However, it will be interesting to see what the application and development of optical spectroscopy approaches that are sensitive to such phase transitions, such as Brillouin light scattering (Bailey et al., 2020; Krüger et al., 1980), hold for the future of investigating these mechanisms. Finally, the plant data discussed in this Review is largely derived from studies in *Arabidopsis*. In future studies, it will be interesting to address whether subtle or substantial differences in the endocytosis mechanism exist in other plant species.

CCVs are an exciting area of study wherein one can combine the interdisciplinary expertise from researchers operating in a range of model systems to uncover the details of this essential eukaryotic organelle. Plant CME studies are entering a new phase, as tools now exist to examine it at appropriate resolutions and directly examine the role of potential plant EAP orthologs. Moreover, the proteome of plant CCVs has now been defined (Dahhan et al., 2022), and we are beginning to uncover unique mechanisms at the molecular scale.
